# Apatinib Monotherapy for Chemotherapy-Refractory Metastatic Colorectal Cancer: A Multi-centre, Single-Arm, Prospective Study

**DOI:** 10.1038/s41598-020-62961-5

**Published:** 2020-04-08

**Authors:** Fen Wang, Xia Yuan, Jun Jia, Xiaoxia Bi, Zeqiang Zhou, Qiming Zhou, Xia Li, Changguo Luo, Minghui Deng, Liangjie Yi, Yong Li, Jianxin Lu, Wenzhi Su, Hanbin Chen, Yu Zhu, Shubin Wang

**Affiliations:** 1 Department of Oncology, Peking University Shenzhen Hospital, Guangdong, China; 2 Department of Oncology, Huizhou Municipal Central Hospital, Guangdong, China; 3 Department of Oncology, Dongguan People’s Hospital, Guangdong, China; 4 Department of Oncology, Huizhou First People’s Hospital, Guangdong, China; 5 Department of Oncology, Shenzhen Second People’s Hospital, Guangdong, China; 6 Department of Oncology, Shenzhen Nanshan People’s Hospital, Guangdong, China; 7 Department of Oncology, Longgang Central Hospital of Shenzhen, Guangdong, China; 8 Department of Oncology, Baoan District Traditional Chinese Medicine Hospital of Shenzhen, Guangdong, China; 9 Department of Oncology, Huizhou Sixth People’s Hospital, Guangdong, China; 10 Department of Oncology, Huizhou Traditional Chinese Medicine Hospital, Guangdong, China; 11 Department of Oncology, Guangdong Hospital of Traditional Chinese Medicine, Guangdong, China; 12 Department of Oncology, People’s Hospital of Shanwei, Guangdong, China; 13 Department of Oncology, Second People’s Hospital of Shanwei, Guangdong, China; 14 Department of Oncology, Pengpai Memorial Hospital of Haifeng, Guangdong, China; 15 Shenzhen Peking University-Hongkong University of Science and Technology Medical Center, Guangdong, China

**Keywords:** Tumour angiogenesis, Targeted therapies

## Abstract

Angiogenesis inhibitors are of considerable interest for treating metastatic colorectal cancer (mCRC). This trial evaluated the efficacy and safety of apatinib in chemotherapy-refractory mCRC. Apatinib 500 mg was administered daily to patients who had progressed after two or more lines of standard fluorouracil-based chemotherapy. Primary endpoint was progression-free survival (PFS). Secondary endpoints were objective response rate (ORR), disease control rate (DCR), overall survival (OS), and toxicity. Overall, 48 patients were enrolled. ORR and DCR were 8.3% (4/48) and 68.8% (33/48), respectively. Median PFS and OS were 4.8 (95% confidence interval [CI], 3.653–5.887) and 9.1 months (95% CI, 5.155–13.045), respectively, and did not differ between subgroups stratified by previous anti-angiogenic therapies. The most prevalent grade 3–4 adverse events were hypertension (12.5%), hand-foot syndrome (HFS, 10.4%), thrombocytopenia (10.4%), and proteinuria (8.3%). Low baseline neutrophil/lymphocyte ratio (NLR, hazard ratios [HR], 0.619; P = 0.027), early carbohydrate antigen 19–9 (CA19–9) decrease (HR, 1.654; P = 0.016), and HFS (HR, 2.087; P = 0.007) were associated with improved PFS. In conclusion, apatinib monotherapy demonstrated encouraging efficacy with manageable toxicities in chemotherapy-refractory mCRC. Previous anti-angiogenic therapies did not influence outcomes. Baseline NLR, early CA19-9 decrease, and HFS could predict the efficacy of apatinib.

## Introduction

Colorectal cancer (CRC) remains the third leading cancer globally^1^, and approximately 40–50% patients present with advanced disease at diagnosis^2^. Until recently, the standard of care for patients in this setting has been regorafenib or trifluridine/tipiracil (TAS102) after the progression of fluoropyrimidine-based chemotherapy with or without targeted therapy^3–7^. Unfortunately, despite these treatments, overall survival (OS) of this population is poor with a <12% survival rate at 5 years^2^.

A substantial proportion of patients have been noted to retain a relatively good performance status after previous standard chemotherapy, which motivates them to undergo further therapy. Because of the unavailability of regorafenib and TAS102 in China at a certain time, numerous exploratory trials have evaluated oxaliplatin-reinduction chemotherapy and salvage chemotherapy with new combinations^3–6^. However, the efficacy of subsequent chemotherapies has been discouraging. This has raised the possibility that other vascular epidermal growth factor receptor (VEGFR) inhibitors, with similar mechanism of activity as regorafenib, could be potential options for the treatment of metastatic CRC (mCRC) patients, who failed to respond to standard chemotherapies.

Apatinib is an orally bioavailable tyrosine kinase receptor (TKI) that selectively inhibits VEGFR-2, including anti-proliferation and anti-angiogenic response^7,8^. It is currently approved by China Food and Drug Administration (CFDA) for treatment in the third-line settings in the patients with metastatic gastric or gastroesophageal junction adenocarcinoma. Preclinical and clinical trials have shown its vigorous antitumour activity and good tolerability in multiple malignancies including non-small cell lung cancer, triple-negative breast cancer, ovarian cancer, and colorectal cancer^7,9–20^, Presently, the evidence that apatinib may improve survival in chemotherapy-refractory mCRC is based on limited retrospective studies^10,13,15,16^. The impact of previous anti-angiogenic therapies on the efficacy of apatinib remains unknown.

Therefore, we designed this single-arm, prospective study to evaluate the efficacy and safety of apatinib monotherapy for mCRC patients who had not responded to standard chemotherapies. The effect on overcoming the resistance of previous anti-angiogenic agents and potential predictive and prognostic factors of apatinib were further investigated.

## Results

### Patient characteristics

**Figure 1:**
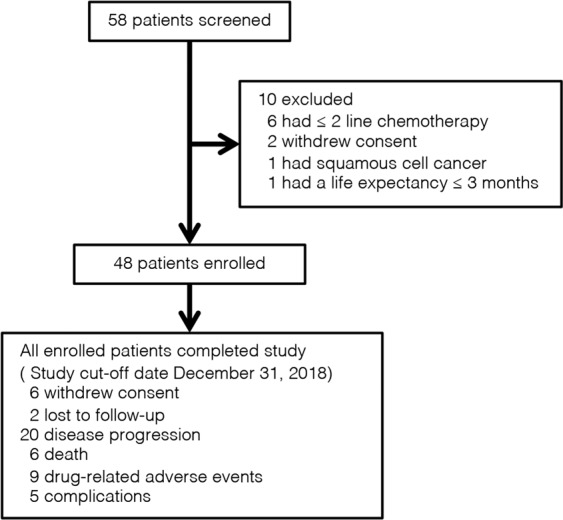
CONSORT diagram of study population selection for chemotherapy-refractory metastatic colorectal cancer.

From 18 April 2017 to 18 October 2018, 58 patients were screened and 48 were accrued **(**Fig. 1**)**. As of the data cut-off date of 31 December 2018, the median follow-up time was 10.3 months (3.0–17.6). Of the 48 enrolled patients, 20 (41.7%) withdrew from the study because of disease progression, including 7 (35%), who experienced dose reduction. Apatinib was also discontinued because of drug-related adverse events (AEs; nine [18.8%]), consent withdrawal (six [12.5%]), loss to follow-up (two [4.2%]), complications (five [10.4%]), and death (six [12.5%]).

Baseline demographics and pre-treatment characteristics are shown in Table 1. The median age was 55 (26–81) years and approximately half of the patients were male (25 [52.1%]). Two-thirds of the patients had an Eastern Cooperative Oncology Group (ECOG) performance status of 0–1 (31 [64.6%]). Most patients had multiple metastases (39 [81.2%]), and the liver was the most common metastasis site (35 [72.9%] patients), while 31 (64.6%) had not been previously treated with any biological targeted therapy before enrolment. Twenty-three (47.9%) patients had previously received three or more lines of treatment for mCRC, including TAS102 (2.1%), other platinum agents (4.2%), raltitrexed (14.6%), fruquintinib (4.2%), S-1 (18.8%), and mitomycin (4.2%). The median neutrophil/lymphocyte ratio (NLR) was 4.1 (2.3–9.8). The median carcinoembryonic antigen (CEA) and carbohydrate antigen 19–9 (CA19–9) were 143.6 ng/mL and 190.7 U/mL, respectively.

**Table 1:** Patients baseline characteristics (N = 48). ECOG, Eastern Cooperative Oncology Group; IQR, inter quartile range; VEGF, vascular endothelial growth factor; EGFR, epidermal growth factor receptor; NLR, neutrophil/lymphocyte ratio; LDH, lactate dehydrogenase; CEA, carcinoembryonic antigen; CA19-9, carbohydrate antigen 19-9.

Characteristics	No. (%)
Median age, years (range)	55 (26–81)
Sex	
Men	25 (52.1)
Women	23 (47.9)
ECOG performance status	
0–1	31 (64.6)
≥ 2	17 (35.4)
Primary tumor location	
Left	34 (70.8)
Right	12 (25.0)
Unknown	2 (4.2)
Differentiation	
Well	6 (12.5)
Moderate	28 (58.3)
Low	14 (29.2)
KRAS mutation	
No	8 (16.7)
Yes	2 (4.2)
Unknown	38 (79.2)
Number of metastatic sites	
Single	9 (18.8)
Multiple	39 (81.2)
etastatic site	
Lung	24 (50.0)
Liver	35 (72.9)
Peritoneum	15 (31.3)
Ovary	7 (14.6)
Liver metastases	35 (72.9)
Synchronous	28 (80.0)
Metachronous	7 (20.0)
Number of previous systemic chemotherapy	
2	25 (52.1)
≥3	23 (47.9)
Previous targeted therapy	
Neither	31 (64.6)
Both anti-VEGF and anti-EGFR	2 (4.2)
Anti-VEGF only	13 (27.1)
Anti-EGFR only	2 (4.2)
Laboratory index, median (range)	
NLR	4.1 (2.3–9.8)
Albumin, g/dl	41.7 (30.3–48.6)
LDH, U/L	287.5 (79.0–1619.0)
CEA, ng/ml	143.6 (1.0–2066.0)
CA19–9, U/ml	190.7 (3.0–12000.0)

### Efficacy analysis

Overall, 4, 29, and 8 patients (8.3%, 60.4%, and 16.7%, respectively) achieved partial responses (PR), stable disease (SD), and disease progression (PD). Seven patients went off trial prematurely for assessment. Responding patients (who achieved CR, PR, or SD) received a median of four cycles of apatinib (range, 1–14). The median number of cycles to a first response of PR (n = 4) and SD (n = 29) were 4 (range, 5–14) and 7.5 (range, 1–11), respectively. There was no significant difference in either ORR (P = 0.50) or DCR (P = 0.66) based on previous anti-angiogenic treatment (Table 2).

**Table 2:** Tumour responses in enrolled patients and patients based on prior anti- vascular endothelial growth factor therapies. CR, complete response; PR, partial response; SD, stable disease; PD, progress disease; VEGF, vascular endothelial growth factor.

	No. (%)	Best response cycles Median (Range)	Ever prior anti-VEGF No. (%)	Never prior anti-VEGF No. (%)	P value
Total	48	4.0 (1–14)	15 (31.2)	33 (68.8)	
CR	0 (0)	0 (0)	0 (0)	0 (0)	
PR	4 (8.3)	7.5 (5–14)	2 (13.3)	2 (6.1)	
SD	29 (60.4)	4.0 (1–11)	7 (26.7)	22 (67.7)	
PD	8 (16.7)		3 (20)	5 (15.2)	
Missing efficacy	7 (14.6)		3 (20)	4 (12.1)	
Overall response	4 (8.3)		2 (13.3)	2 (6.1)	0.50
Disease control	33 (68.7)		9 (60)	24 (72.7)	0.66

**Figure 2:**
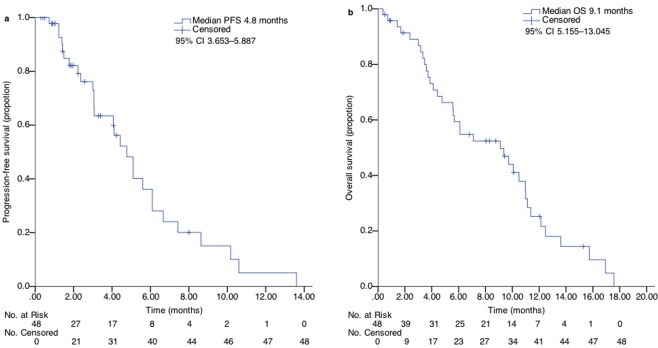
Kaplan–Meier curve of (**a**) PFS and (**b**) OS in the enrolled patients. PFS, progression-free survival; OS, overall survival; CI, confidence interval.

**Figure 3:**
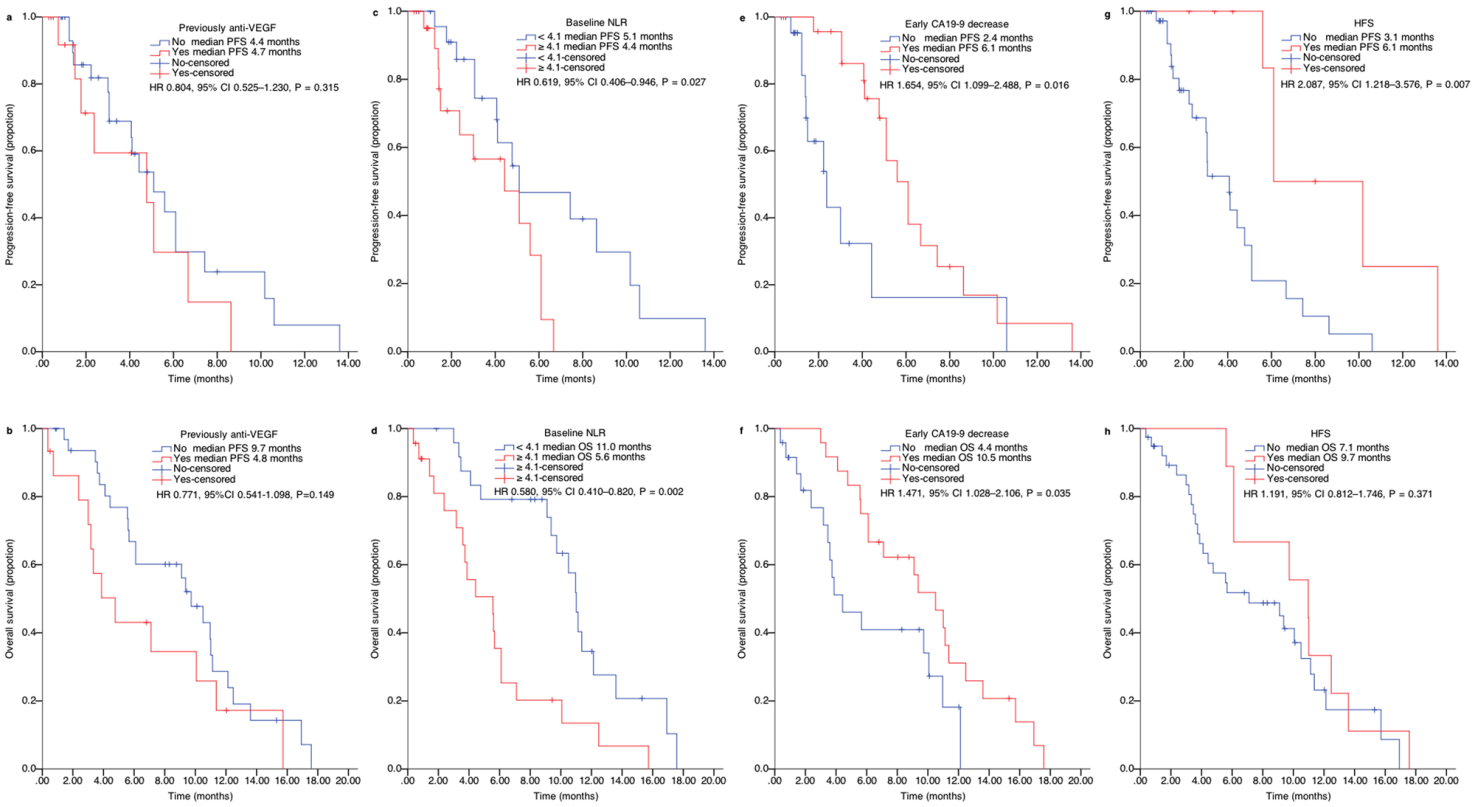
Stratified analysis of progression-free and overall survival by variables. Kaplan–Meier curve of (**a**) PFS and (**b**) OS in patients who were previously treated with or without anti-VEGF therapies, (**c**) PFS and (**d**) OS in patients with different NLR levels, (**e**) PFS and (**f**) OS in patients with or without CA19-9 decrease, and (**g**) PFS and (**h**) OS in patients with or without HFS. PFS, progression-free survival; OS, overall survival; VEGF, vascular endothelial growth factor; NLR, neutrophil/lymphocyte ratio; carbohydrate antigen 19-9, CA19-9; HFS, hand-foot syndrome; HR, hazard ratios; CI, confidence interval.

At data cut-off, all enrolled patients discontinued the treatment and were eligible for survival analysis (Fig. 1) including 15 (31.3%), who had discontinued treatment before progression. After progression or the end of study treatment, 25 (52.1%) patients underwent further systemic anticancer treatment, including 19 (76%), who were naïve to previous anti-angiogenic agents. Median PFS and OS were 4.8 (95% confidence interval [CI], 3.653–5.887) months and 9.1 (95% CI 5.155–13.045) months (Fig. 2a,b), respectively. Those who were naïve to previous anti-angiogenic therapies (n = 33, 68.7%) did not demonstrate a significantly improved PFS (HR 0.804; 95% CI, 0.525–1.230; P = 0.315) or OS (HR, 0.771; 95% CI, 0.541–1.098; P = 0.149) compared to those who were exposed to anti-angiogenic therapies (n = 15, 31.3%), although they seemed to have longer OS (9.7 vs. 4.8 months, Fig. 3a,b).

### Exploratory analysis

**Figure 4:**
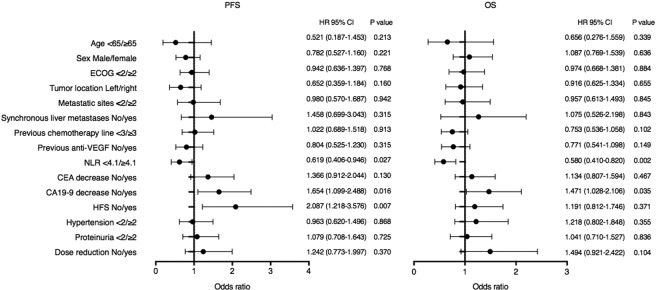
Univariate analysis of factors to predict progression-free and overall survival of apatinib showed by forest plot. PFS, progression-free survival; OS, overall survival; ECOG, Eastern Cooperative Oncology Group; VEGF, vascular endothelial growth factor; NLR, neutrophil/lymphocyte ratio; CEA, carcinoembryonic antigen; CA19-9, carbohydrate antigen 19-9; AE, adverse event; HR, hazard ratios; CI, confidence interval.

The association between clinical outcomes and several variables, including baseline characteristics, laboratory parameters, and drug-related AEs, was analysed in the enrolled patients. Out of forty-eight patients, seven (14.6%) and 11 (22.9%) had initial normal CEA and CA19-9, respectively, and were therefore excluded from the exploratory analysis of tumour markers. The median baseline NLR of 4.1 was adopted as the cut-off value to discriminate the patients with low (NLR < 4.1) versus high (NLR ≥ 4.1) NLR. In general, patient characteristics, including age, sex, ECOG performance status, primary tumour locations, metastatic sites, liver metastases, previous chemotherapy lines, were not associated with differences in PFS and OS (Fig. 4). Low baseline NLR (hazard ratios [HR], 0.619; P = 0.027 and HR, 0.580; P = 0.002) and early CA19-9 decrease (HR, 1.654; P = 0.016 and HR, 1.471; P = 0.035) were significantly associated with improved PFS and OS (Fig. 3c–f). The presence of hand-foot syndrome (HFS) during the first 28 days was associated with longer PFS (HR, 2.087; P = 0.007) but not OS (HR, 1.218; P = 0.355), although a numerical advantage in OS (9.7 vs. 7.1 months) was observed for those who had HFS (Fig. 3g,h**)**. Low baseline NLR (HR, 0.222; P = 0.040) and early CA19-9 decrease (HR, 11.807; P = 0.011) were significantly associated with improved PFS while only low baseline NLR (HR, 0.347; P = 0.003) was significantly associated with improved OS in the multivariate analysis using the above-mentioned factors (Table 3). In addition, NLR was not associated with baseline characteristics, AEs, and apatinib dose modification (Table 4).

**Table 3:** Multivariate analysis of factors to predict progression-free and overall of apatinib. PFS, progression-free survival; OS, overall survival; NLR, neutrophil/lymphocyte ratio; CA19–9, carbohydrate antigen 19–9; HR, hazard ratios.

Variables	PFS		OS	
	HR (95%CI)	P value	HR (95%CI)	P value
NLR				
<4.1/≥4.1	0.222 (0.053–0.935)	0.040	0.347 (0.172–0.702)	0.003
CA19–9 decrease				
No/yes	11.807 (1.771–78.708)	0.011	1.972 (0.909–4.278)	0.086
Hand-foot syndrome				
No/yes	1.119 (0.165–7.737)	0.909	1.164 (0.516–2.625)	0.715

**Table 4:** Correlation between NLR and baseline characteristics, AEs, and apatinib dose modification. NLR, neutrophil/lymphocyte ratio; ECOG, Eastern Cooperative Oncology Group; LDH, lactate dehydrogenase.

Variables, N. (%)	NLR		P-value
	<4.1	≥4.1	
Age			0.140
<65	18 (72.0)	21 (91.3)	
≥65	7 (28.0)	2 (8.7)	
Sex			1.000
Men	13 (52.0)	12 (52.2)	
Women	12 (48.0)	11 (47.8)	
ECOG performance status			1.000
0–1	16 (64.0)	15 (65.2)	
≥ 2	9 (36.0)	8 (34.8)	
Primary tumor location			0.523
Left	17 (70.8)	17 (72.3)	
Right	7 (29.2)	5 (22.7)	
Differentiation			0.416
Well-Moderate	16 (64.0)	18 (78.3)	
Low	9 (36.0)	5 (21.7)	
Number of metastatic sites			1.000
Single	5 (20.0)	4 (17.4)	
Multiple	20 (80.0)	19 (82.6)	
Liver metastases			0.934
Synchronous	14 (77.8)	14 (82.4)	
Metachronous	4 (22.2)	3 (17.6)	
Number of previous systemic chemotherapy			0.386
2	15 (60.0)	10 (43.5)	
≥ 3	10 (40.0)	13 (56.5)	
Albumin			0.772
< 40 g/dl	15 (60.0)	15 (65.2)	
≥40 g/dl	10 (40.0)	8 (34.8)	
LDH			0.259
<287 U/L	12 (48.0)	15 (65.2)	
≥287 U/L	13 (52.0)	8 (34.8)	
Hand-foot syndrome			0.075
No	13 (52.0)	18 (78.3)	
Yes	12 (48.0)	5 (21.7)	
Hypertension			1.000
No	14 (56.0)	13 (56.5)	
Yes	11 (44.0)	10 (43.5)	
Proteinuria			0.401
No	12 (50.0)	14 (63.6)	
Yes	12 (50.0)	8 (36.4)	
Dose modification			0.249
No	19 (76.0)	21 (91.3)	
Yes	6 (24.0)	2 (8.7)	

### Safety analysis

All 48 patients were eligible for safety evaluation. The most prevalent side effects (for all grades) of apatinib were hypertension (43.8%), proteinuria (41.7%), HFS (35.4%), thrombocytopenia (29.2%), neutropenia (27.1%), leukopenia (22.9%), anorexia (22.9%), mucositis oral (22.9%), and fatigue (20.8%). Most of these toxicities were grade 1–2. Five patients experienced ≥ grade 3 thrombocytopenia but none was admitted to the hospital for blood transfusion. One patient had transient grade 3 neutropenia without febrile neutropenia. Six patients experienced ≥ grade 3 hypertension, including one who had a history of severe hypertension and died from hypertensive cerebral haemorrhage after six cycles of apatinib (a serious AE [SAE] was reported). Of the five remaining SAEs possibly related to apatinib recorded in the study, two deaths were related to tumour progression. The other three patients were admitted to the hospital because of perianal abscess and recto-vaginal fistula, which were considered to be related to the treatment, and pathological fracture of L1-2, which was considered to be a disease complication. Dose reductions were required for 8 (16.7%) of the 48 patients, including 7 (87.5%), who only received the first reduction, and 1 (12.5%) who had the second. Dose reductions occurred in 4 (50%) of the 8 patients, who experienced PD. Dose reductions were not associated with outcomes of apatinib in terms of PFS or OS (Fig. 4). The main reasons for dose modification were proteinuria, HFS, and hypertension. The toxicities are summarised in Table 5.

**Table 5:** Summary of toxicities.

Toxicity	No. (%)		
	All grades	Grade 1 to 2	Grade 3 to 4
Fatigue	10 (20.8)	9 (18.8)	1(2.1)
Epistaxis	1 (2.1)	1 (2.1)	0
Gastrointestinal hemorrhage	3 (6.3)	3 (6.3)	0
Liver metastatic lesion hemorrhage	1 (2.1)	0	1 (2.1)
Anorexia	11 (22.9)	11 (22.9)	0
Nausea	7 (14.6)	7 (14.6)	0
Vomiting	6 (12.5)	6 (12.5)	0
Constipation	2 (4.2)	0	0
Abdominal pain	6 (12.5)	5 (10.4)	1 (2.1)
Diarrhea	5 (10.4)	4 (4.2)	1 (2.1)
Mucositis oral	11 (22.9)	8 (16.7)	3 (6.3)
Fistula	1 (2.1)	0	1 (2.1)
Rash	2 (4.2)	2 (4.2)	0
Hand-foot syndrome	17 (35.4)	12 (25.0)	5 (10.4)
Perianal abscess	1 (2.1)	0	1 (2.1)
Proteinuria	20 (41.7)	16 (33.3)	4 (8.3)
Anemia	4 (8.4)	4 (8.4)	0
Neutropenia	13 (27.1)	12 (25.0)	1 (2.1)
Thrombocytopenia	14 (29.2)	9 (18.8)	5 (10.4)
Leukopenia	11 (22.9)	9 (18.8)	1 (2.1)
ALT elevation	6 (12.5)	5 (10.4)	1 (2.1)
AST elevation	9 (18.8)	7 (14.6)	2 (4.2)
Hyperbilirubinemia	10 (20.8)	8 (16.7)	2 (4.2)
Hypokalemia	1 (2.1)	1 (2.1)	0
Headache	3 (8.4)	3 (8.4)	0
Dizziness	2 (4.2)	2 (4.2)	0
Trachyphonia	1 (2.1)	1 (2.1)	0
Hypertension	21 (43.8)	15 (31.3)	6 (12.5)^a^
Hypothyroidism	1(2.1)	1 (2.1)	0

^a^One patient had a severe hypertension and died from hypertensive cerebral hemorrhage.

## Discussion

This study of apatinib monotherapy for chemotherapy-refractory mCRC met its primary and secondary endpoints of improving survival outcomes over the historical control expected from placebo or best supportive care (BSC). The historical estimate for median PFS and OS of placebo or BSC as ≥ third-line therapy in mCRC are 1.7 and 6.3 months, respectively^21,22^, and apatinib improves on these values with median PFS and OS of 4.8 and 9.1 months, respectively.

Multiple randomised controlled trials (RCTs) have been conducted to investigate the role of third- or later-line therapies with small-cellular multi-kinase inhibitors in a similar patient population. PFS and OS appear to be slightly improved by regorafenib above BSC (median PFS and OS, 1.9 vs. 1.7 and 6.4 vs. 5.0 months, respectively) or placebo (median PFS and OS, 3.2 vs. 1.7 and 8.8 vs. 6.3 months, respectively) in the CORRECT and CONCOUR studies^21,23^. Another TKI famitinib demonstrated similar survival benefit, with PFS and OS of 2.0 and 6.0 months, respectively. However, the absolute survival benefit of regorafenib and famitinib remains limited. Recently, fruquintinib was accepted as an alternative third- or later-line therapy in China based on a superior survival to that of the placebo, with a PFS and OS of 3.7 and 9.3 months, respectively^24^.

In our study, apatinib demonstrated comparable survival outcomes. The observed efficacy of apatinib may be attributable to following reasons. First, apatinib selectively targets VEGFR-2, which binds to all the VEGF-A isoforms, VEGF-C, and VEGF-D to down-regulate VEGF-mediated endothelial cell proliferation, migration, and tumour microvascular density^25,26^. Several studies have confirmed VEGFR-2 as the key receptor in angiogenesis^27,28^. Second, apatinib mediates the regulation of autophagy and apoptosis of colon cancer cell by inhibiting the AKT-mechanistic target of rapamycin kinase (mTOR) signalling pathway and increasing the expression of light chain 3 (LC3)-II, which is a marker for the autophagosome number^29^. Third, preclinical studies have proven that prolonged exposure to anti-VEGF treatment beyond the discontinuation of cytotoxic agents may improve tumour control by delaying progression in xenograft models of CRC^30^. This is consistent with the clinical benefit of bevacizumab in cross-line therapy with an accompanying change in the cytotoxic chemotherapy backbone, implying that the development of resistance is often chemotherapeutic agent-specific. Furthermore, the success of continuing of therapy with anti-angiogenic agents such as regorafenib and fruquintinib (currently approved by the CFDA) in later-line therapy also indicates that anti-angiogenesis are becoming integral to the whole-process management of mCRC^17,21,22^. Lastly, there are potentially important biological differences between anti-angiogenic agents. Direct targeting of the receptor, as opposed to the ligand, might overcome the resistance to anti-angiogenic therapies such as bevacizumab^31^.

Our results are consistent with those of recently published studies of apatinib in a similar population of patients with mCRC conducted by Guo *et al*., (PFS 3.8 months, OS not reached)^10^; Liang *et al*., (PFS 4.8 months, OS 10.1 months)^15^; and Li *et al*., (PFS 3.7 months, OS 7.3 months)^13^. This finding further supports the use of apatinib as an alternative option based on the strategy of continued administration of newer anti-angiogenic agents as the new standard of care. In a retrospective case-controlled study, apatinib exhibited weaker survival outcomes with PFS and OS of 2.0 and OS 5.0 months, respectively, in late-line setting in mCRC^16^. We speculated that the inconsistency could be because the previous study had a relatively shorter follow-up time (6.0 months), higher proportion of patients with performants status of 2 (48.1%), smaller sample size (25 patients in apatinib group), and higher selection bias than those of our present study.

There are points to consider when interpreting the results. However, the proportion of patients previously exposed to anti-VEGF treatment may have arguably contributed to the apparent improvement in patient outcomes in our study. Indeed, in contrast to North America and Europe, in China the targeted therapy including anti-angiogenic agents are not routinely included in the initial treatment patterns in mCRC at the time of this study. In our trial only 31.3% of patients, as opposed to 41% and 100% in the CONCOUR and CORRECT trials, respectively, had been previously treated with VEGF inhibitors. However, our pre-set stratified analysis showed no significant differences in OS and PFS based on exposure to VEGF-targeted therapies before the trial, suggesting that the efficacy of apatinib may not be affected by previous anti-angiogenic treatments.

Despite of this exploratory analysis of the small sample size in subgroup, our findings are in accordance with those of the FRESCO study. In the FRESCO study, a similar proportion of patients were observed to have been exposed to VEGF inhibitors (approximately 30% in the fruquintinib and placebo groups) and fruquintinib still demonstrated survival benefits in the ever-anti-VEGF subgroup over compared with the placebo (HR, 0.68; 95% CI, 0.45–1.03])^24^. These findings provide clinical evidence that different anti-angiogenic agents with varying anti-tumour mechanisms have no complete cross-resistance. A numerical OS advantage was observed with those who were not previously treated with anti-angiogenic agents compared with those who were previously treated, although the difference was not significant. A higher proportion of patients exposed to subsequent systemic anticancer therapy in the naïve-to-anti-angiogenic agent-treated group (76% vs. 24%) might have contributed to the numerical OS advantage in our study. A conflicting result was reported for prior anti-angiogenic therapy as an independent factor associated with the PFS and OS of apatinib in mCRC in a real-world observation study^13^. However, the difference in study design, where >40% of patients received apatinib co-therapy in that study, makes the results incomparable.

In contrast, more than one-third of patients had a PS ≥ 2 and more than 40% had received three or more lines of systemic chemotherapies, indicating apatinib was administered to a heavily pre-treated population with poor prognosis in this study. The efficacy of apatinib may be underestimated because of the shorter life expectancy and poor conditions. The constant development of newer anti-angiogenic agents for mCRC makes it important to determine their most appropriate and rational place in the treatment arsenal^31^.

The AEs observed in our study were either similar to those reported in previous studies of apatinib^10–12,19,20,32–35^ or to those of other VEGFR inhibitors^24,36,37^. Most grade 3 or 4 AEs were hypertension, proteinuria, HFS, thrombocytopenia, and mucositis oral, which was expected and tolerable for this patient population. Toxicities can be well-managed using symptomatic treatments and dose modification, suspension, or termination. Apatinib was started at 500 mg in our study, rather than the 850 mg dose reported in previous studies of gastric cancer^33^. This initial dose and the allowance of two dose reductions in our study were based on the following considerations.

First, by the time patients have progressed to a third-line treatment, they have received considerable chemotherapy and their tolerability to treatments may have been impaired. Second, patients who received a dose of apatinib <850 mg in previous studies experienced less toxicity^33,34^. Third, apatinib presented significant interpatient variability in a phase I study, indicating that dose modification is warranted to meet individual needs^7^. The moderate initial dose in our study resulted in a more manageable toxicity profile and a lower proportion of dose modification than those in the previous studies of apatinib 850 mg in advanced gastric cancer^33,34^. Furthermore, it seems comparable to the modification reported in the studies of apatinib 500 mg in mCRC^10,13–16^.

Of note, angiogenic TKIs do not all have same AE profiles. In the CONSIGN study, the most frequent ≥ grade 3 AEs that were associated with regorafenib were fatigue, HFS, and hypertension (13%, 14%, and 15%, respectively)^38^. These varied toxicity profiles could potentially aid patients who have contraindications to one anti-angiogenic agent to switch to other drugs in the same class.

It has been extremely challenging to identify suitable predictive and prognostic markers of anti-VEGF agents^39^. In our exploratory analysis, low baseline NLR, CA19-9 decrease, and HFS during the first cycle of apatinib were associated with statistically significant and clinically meaningful improvement in survival.

NLR, as a laboratory marker of systemic inflammatory response, has been studied as a potential prognostic and predictive factor for several tumours including CRC. Two previous studies have demonstrated the predictive and prognostic role of NLR in bevacizumab-containing chemotherapy and apatinib in mCRC, respectively, with a negative impact on PFS and OS^13,40^. Likewise, our study showed NLR negatively predicted the outcome of apatinib and was an independent prognostic factor in mCRC. The more widely used cut-off range of 3 to 5^41,42^ was considered when setting the median NLR cut-off value of 4.1 in the present study. Why high NLR predicted a worse efficacy for apatinib has remained unidentified. We hypothesised that the extra VEGF secreted by the more abundant neutrophils may interfere with the inhibition of tyrosine kinase of VEGFR2 by apatinib and bind with VEGFR1, thus activating the VEGF signal pathway^43,44^. In addition to VEGF, a variety of other carcinogenic factors overproduced by elevated neutrophils, such as neutrophil elastase and matrix metalloproteinase-9, participate in extracellular matrix remodelling and in tumour angiogenesis and growth^45,46^. Conversely, lymphocytopenia, as an important component of high NLR, can lead to a decreased level of tumour-infiltrating lymphocytes, which is regarded as a muted or absent anticancer immunological reaction associated with worse efficacy and survival^47,48^. Therefore, an imbalance between inflammation (promoting tumour) and immunity (defending tumour) reflected in the high NLR^49^ may explain its prognostic role in the present study. In addition, high NLR is associated with a more aggressive tumour phenotype in resectable CRC^50,51^, and the greater magnitude of the systemic inflammatory response is associated with poorer nutritional/functional status of the host, which might weaken the treatment tolerance and compliance in cancer patients^52^, both of which ultimately leading to worse survival. However, no significant associations were observed between NLR and tumour features and apatinib tolerance and compliance (as evidenced by AEs and dose modification) in the present study. Thus, NLR may provide prognostic information independently of the known prognostic factors in chemotherapy-refractory patients with mCRC receiving apatinib. It should be noted that concomitant conditions, such as infections or inflammation could influence neutrophil and lymphocyte counts^53^. The strict inclusion and exclusion criteria and prospective collection of patient data in our study would avoid these potential effects and enhance the reliability of our results.

Early decrease of CA19-9, rather than CEA, demonstrated a significantly positive prediction efficiency in our study. Indeed, CEA is a more commonly used tumour marker than CA19-9 given the insufficiency of the latter in monitoring systemic chemotherapy for mCRC^54^. The association of CA19-9 with the efficacy of apatinib has not been reported but there is evidence in similar biomarker studies of other anti-angiogenic agents. Levels of or changes in CA19-9 have been reported to predict the efficacy of bevacizumab or bevacizumab-containing chemotherapy and regorafenib in mCRC in some previous studies^55–57^. We have suggested two possible reasons why CA19-9 rather than CEA was an early predictive factor in our study. First, CA19-9 acts as an adhesion factor in vascular endothelial cells via secretin^58^ and when apatinib blocks VEGF signalling, inhibition of secretin release decreases CA19-9. Second, the shorter half-life of CA19-9 may contribute to an early response to antitumour therapy. Elevated basal level of CA19-9 has been widely accepted as a prognostic factor in CRC^59^. Other studies have suggested baseline and dynamic change of tumour markers to be independent prognostic factors in mCRC^60,61^. In the present study, the prognostic effect of better OS for the early decrease of CA19-9 was observed in univariate analysis. This might be due to a slower marker production owing to the reduced tumour burden and relatively lower biological aggression of tumour cells after effective treatment^61,62^. However, changes in CA19-9 failed to be an independent prognostic factor in multivariate analysis. Subsequent systemic anticancer treatment, which almost half of the patients underwent in our study, might influence evaluation of the relationship between OS and CA19-9 decrease.

Treatment-related AEs has been identified as a viable biomarker of the efficacy of anti-VEGF agents in several solid tumours including CRC^63–66^. An analogous positive relationship between the presence of HFS during the first cycle of apatinib and improved survival was observed in our study. The intrinsic mechanisms of HFS caused by blocking of the VEGF pathway have not been elucidated. HFS is considered to be caused by reduced vascular reconstruction in healthy skin tissue after blocking VEGF, and is known to have a dose-dependent relationship with the agents^67^. Therefore, the presence of HFS may partly reflect an effective inhibition of the VEGF pathway and thus predict the efficacy of anti-VEGF agents. Nevertheless, the presence of HFS may also result from pharmacokinetic differences in host individuals, so a mechanism other than VEGF inhibition cannot be ruled out. The relationship between HFS and survival was not confirmed by multivariate analysis in the present study. We believe it would be better evaluated in a larger group of patients as the occurrence rate of HFS has to be considered.

The results from multivariate analysis need to be interpreted with caution. The missing values for the early decreased CA19-9 would affect the significance of the results. Moreover, it is reasonable to consider that the decrease in CA19-9 and presence of HFS are not as excellent as the pre-existing inherent biomarkers because they appear after the treatment. However, this is an exploratory analysis and the sample size was not calculated based on the endpoint of biomarkers study. In addition to the evidence from studies, these markers are readily available, easily measurable, occur early, and are inexpensive in diagnosing and monitoring mCRC. If the predictive and prognostic values are validated in subsequent studies, these markers could be surrogate indicators to predict the efficacy of apatinib and serve as supplemental indexes to increase the prognostic accuracy in mCRC.

There are a few limitations to the utility of our current study. First, two-thirds of the patients did not receive molecular target drugs in prior treatments, which deviated from the standard of care based on the worldwide consensus. Nevertheless, the best strategy for utilising agents with different mechanisms of action in mCRC is still undecided. Apatinib may be an attractive alternative as a non-myelotoxic option for patients who have exhausted their bone marrow reserve after progression of multi-lines chemotherapy. Moreover, apatinib provides additional benefits by exposing CRC cells to a very different anti-tumour mechanism, and therefore, it may affect the efficacy of subsequent treatments. The second limitation was the failure to identify the KRAS mutation status of most patients, which predicts the efficacy of epidermal growth factor receptor (EGFR)-targeted therapy and prognosis of CRC. Nevertheless, KRAS mutation status identification is more critical in selecting EGFR-targeted than VEGF-targeted therapies. Therefore, our study may have only lost information on the prognostic value of KRAS mutation status, which has been illuminated clearly. In addition, single-arm design, small sample size, and non-diverse Chinese population may also affect the objectivity of our data.

Despite these limitations, our study has several advantages. First, it is a rigorously performed prospective study with independent monitoring and a centralised review of the radiological responses, which minimises chances for error and bias. Second, we further explored the impact of previous anti-angiogenic agents and potential predictive and prognostic factors of apatinib in mCRC, which is a scientifically valid contribution that offers clues to further studies of predictive and prognostic markers of anti-angiogenesis therapy.

## Methods

### Study design and procedures

This was a single-arm, prospective study (Chinese Clinical Trial Registry, ChiCTR1900020503) conducted at 14 centres in China, was approved by the institutional review boards of each of participating centre. The study was conducted in accordance with provisions of the Declaration of Helsinki and Good Clinical Practice. All subjects provided informed consent prior to participating in the study.

Apatinib was administered at a daily dose of 500 mg on a 28-day cycle. The treatment course was continued until tumour progression, unacceptable toxicity, consent withdrawal or cessation by the physicians’ decision. Dose reductions to 250 mg once daily (first reduction) and 250 mg once every 2 days (second reduction) were allowed for drug-related toxicity. Apatinib was suspended until the toxicity was resolved to < grade 1 (for grade 3/4 non-haematological toxicities) or grade 2 (for grade 3/4 haematological toxicities). Then the drug was re-administrated at the same dosage (grade 3) or with one-level reduction (grade 4). Where drug-related toxicity persisted despite treatment interruption after 2 weeks or the patient failed to meet the criteria defined in the protocol despite two dose reductions, apatinib was discontinued permanently.

### Patient selection

Patients >18 years old with a histological confirmation of colorectal adenocarcinoma and who experienced a relapse with failure of standard chemotherapies including fluoropyrimidine, oxaliplatin, and irinotecan were eligible for the study. Failure of standard treatment was determined by two criteria: (i) documented radiological deterioration during or within 3 months from the last cycle of front-line treatment with fluoropyrimidine-based chemotherapies (or within half a year from completion of adjuvant oxaliplatin) administered and (ii) halting of previous treatment due to intolerable toxicities before enrolment. Patients previously treated with targeted monoclonal antibodies were also included. Previous bevacizumab administration was one of the stratification factors used to analyse the efficacy of apatinib. Other eligibility criteria were radiologically measurable disease as assessed using Response Evaluation Criteria in Solid Tumours (RECIST, version 1.1)^68^; performance status of 0 to 3 for ECOG; a minimum of 3-month life expectancy; and adequate haematologic, hepatic, and renal function.

Patients with uncontrolled blood pressure, severe proteinuria (urine protein ≥+++ or>1.0 g over 24 hours, or both), severe impairment of gastrointestinal absorption, evidence of active bleeding or bleeding diathesis, symptomatic central nervous system metastases, history of severe cardiovascular diseases, and concurrent severe and/or uncontrolled medical disease were excluded from the study. Full inclusion and exclusion criteria are documented in the supplementary file 1.

### Outcomes

The primary endpoint was PFS, defined as the time from the date of the first drug administration to the date of documentation of disease progression or death from any cause. Secondary endpoints were OS (time from the first drug administration to death or last follow-up), ORR, DCR, and toxicity (as graded using the National Cancer Institute-Common Terminology Criteria for Adverse Events [NCI-CTCAE, version 4.03]).

### Patient evaluation

Patients underwent continuous investigation during the entire process of study. Prior to each cycle, complete medical record documentation and physical examinations were performed. Radiological examinations (computed tomography [CT] or magnetic resonance imaging [MRI]) were performed at baseline and every 4 weeks during the first cycle and every 8 weeks afterwards.

The efficacy evaluation complied with either RECIST1.1 or was based on the clinical discretion of the investigators where patients were unable to undergo imaging examinations (e.g. Because of deterioration of condition). Confirmation of responses were required.

Safety assessments such as AEs, specialised examinations (electrocardiograms and echocardiographs), and laboratory abnormalities (clinical chemistry, urinalysis, and haematology) were included. Before each cycle, once a week during the first cycle, and every 2 weeks during the second cycle, the complete blood count (CBC) and blood chemistry analysis were performed. Treatment-related AEs were categorised and graded according to the NCI-CTCAE (version 4.03). After treatment completion, follow-up was conducted for a maximum of 3 months.

### Laboratory data collection

The following laboratory data were collected from the medical records of patients: biochemical parameter values (albumin, LDH, CEA, and CA19-9) and blood cell counts (leukocytes, neutrophils, lymphocytes, monocytes, and platelets) at baseline. CEA and CA19-9 were re-collected at day 28 (from 7 to 28 days) after apatinib administration. Tumour marker decrease was defined as an abnormal parameter with a decrease >10%. Patients with normal initial tumour markers were not considered. The NLR was defined as the absolute neutrophil count divided by the absolute lymphocyte count.

### Statistical analysis

The design of this study was a one-sided with an α-error of 5% and power of 90%. Previous studies reported the median PFS of the placebo or BSC with ≥3rd line therapy in mCRC was 1.7 months^21,22^. We expected that the median PFS of apatinib would be 3 months (n = 34). The final patient accrual number was 40 under the assumption of an 18-month accrual period, 6-month follow-up period, and 20% dropout rate.

Baseline demographic and disease characteristics were summarised as medians (range) for continuous variables and proportions for categorical variables. The Kaplan–Meier method was used to analyse the PFS and OS. The log-rank test and Cox regression models were used for univariate and multivariate analysis to correlate previous anti-VEGF treatment and other variables of interest with the PFS and OS, respectively. Variables with P-values < 0.10 in univariate analysis were set as covariates in the multivariate analysis; P-values < 0.05 were considered statistically significant. All statistical analysis were conducted using the statistical package for the social sciences (SPSS) version 16.0 (SPSS Inc., Chicago, IL, USA). The overall credibility of the study and safety of the participants were ensured by the supervision of an independent committee, consisting of one statistician and three oncologists.

### Ethical approval and informed consent

The study was approved by the institutional review board of Peking University Shenzhen Hospital and the Ethics Committees of each centre before the initiation of the study and conducted in accordance with the provisions of the Declaration of Helsinki and Good Clinical Practice. Informed consent was obtained from all participants or their legal guardians. This study was retrospectively registered with the Chinese Clinical Trial Registry on 03/01/2019, number ChiCTR1900020503. The Chinese Clinical Trial Registry critically reviewed the data and documents and then assigned the authorised clinical trial number after all the patients’ information and material were uploaded.

## Conclusions

To the best of our knowledge, this is the first prospective study to evaluate the efficacy and safety of apatinib and explore the impact of previous anti-angiogenic agents and predictive factors on its efficacy in chemotherapy-refractory mCRC. Apatinib monotherapy demonstrated encouraging efficacy with manageable toxicities in this setting. This efficacy was not affected by previous administration of anti-angiogenic therapies. Clinical parameters including baseline NLR, early CA19-9 decrease and HFS could be predictive factors of the efficacy of apatinib.

## Supplementary information

Supplementary Information.

## Data Availability

The raw data are not publicly available due to internal policy but are available from the corresponding author on reasonable request.
