# Supplementary material for: Apatinib Monotherapy for Chemotherapy-Refractory Metastatic Colorectal Cancer: A Multi-centre, Single-Arm, Prospective Study
Source: Sci Rep. 2020 Apr 8;10:6058. doi: 10.1038/s41598-020-62961-5 (PMC7142071; doi:10.1038/s41598-020-62961-5)
Supplement: Supplementary file 1 — Supplementary Information. [file 41598_2020_62961_MOESM1_ESM.pdf]

SUPPLEMENTARY MATERIAL

Apatinib Monotherapy for Chemotherapy-Refractory Metastatic Colorectal Cancer: A Multi-centre, Single Arm, Prospective Study

Fen Wang MD[1] | Xia Yuan MD[2] | Jun Jia MD[3] | Xiaoxia Bi MD[4] | Zeqiang Zhou MD[5] | Qiming Zhou MD[6] | Xia Li MD[7] | Changguo Luo MD[8] | Minghui Deng MD[9] | Liangjie Yi MD[10] | Yong Li MD[11] | Jianxin Lu MD[12] | Wenzhi Su MD[13] | Hanbin Chen MD[14] | Yu Zhu PhD[1,15] | Shubin Wang MD, PhD[1,15*]

[1]Department of Oncology, Peking University Shenzhen Hospital, Guangdong, China

[2]Department of Oncology, Huizhou Municipal Central Hospital, Guangdong, China

[3]Department of Oncology, Dongguan People's Hospital, Guangdong, China

[4]Department of Oncology, Huizhou First People's Hospital, Guangdong, China

[5]Department of Oncology, Shenzhen Second People's Hospital, Guangdong, China

[6]Department of Oncology, Shenzhen Nanshan People’s Hospital, Guangdong, China

[7]Department of Oncology, Longgang Central Hospital of Shenzhen, Guangdong, China

[8]Department of Oncology, Baoan District Traditional Chinese Medicine Hospital of Shenzhen, Guangdong, China

[9]Department of Oncology, Huizhou Sixth People's Hospital, Guangdong, China

[10]Department of Oncology, Huizhou Traditional Chinese Medicine Hospital, Guangdong, China

[11]Department of Oncology, Guangdong Hospital of Traditional Chinese Medicine, Guangdong, China

[12]Department of Oncology, People's Hospital of Shanwei, Guangdong, China

[13]Department of Oncology, Second People's Hospital of Shanwei, Guangdong, China

[14]Department of Oncology, Pengpai Memorial Hospital of Haifeng, Guangdong, China

[15]Shenzhen Peking University-Hongkong University of Science and Technology Medical Center, Guangdong, China

***Corresponding author:** Shubin Wang, M.D., Ph.D., Department of Oncology, Peking University Shenzhen Hospital, No. 1120 Lianhua Road, Futian District, Shenzhen, Guangdong, China, 518036. +8613823394076; pkuszh_oncology@163.com

SUPPLEMENTARY FILE 1

Inclusion Criteria and Exclusion Criteria

Inclusion Criteria

1. Male or female patient aged 18 years old; 2. Patients with advanced nodular and rectal adenocarcinoma (all other histological types excluded) confirmed by pathology (see Annex I); 3. Previously received ≥ second-line standard chemotherapy and treatment failed; Definition of treatment failure: (1) Disease progression during treatment or disease progression within 3 months after the last treatment, with clear evidence of imaging or clinical progression; (2) Exit criteria due to inability to tolerate chemotherapy adverse events. The patients treated, according to the CTCAE 4.0 standard, the severity level of intolerable hematologic adverse events needs to reach above IV (platelet decline is above III) or non-hematologic adverse events reach grade III or above, and the researchers judge The subject was still unable to tolerate repeating the original regimen; 4. According to the RECIST 1.1 standard, the patient has at least one target lesion with a measurable diameter (CT scan with a long diameter of ≥10 mm, CT scan with a short diameter of ≥15 mm, and a scan layer thickness of no more than 5 mm; Over-the-counter treatment); 5. ECOG physical status score 0~3 points (see Annex 2); 6. The estimated survival period is ≥ 3 months; 7. The main organs function well, that is, the following requirements are met one week before enrollment: (1) Blood routine examination: Hemoglobin > 80 g/L (no blood transfusion within 14 days); Neutrophil count $>1.5\times10^{9}$/L; Platelet count $>80\times10^{9}$/L; (2) Biochemical examination: Total bilirubin ≤ 1.5 × ULN (upper normal limit); ALT or ALT ≤ 5 ×

ULN; Endogenous creatinine clearance ≥ 50 ml/min (Cockcroft-Gault formula); 8. Subjects received damage from other treatments, including nitroso or mitomycin at odds of ≥6 weeks; other cytotoxic drugs, radiation or surgery ≥4 weeks, and wounds Completely healed; 9. Sign the informed consent form; 10. Compliance is good, family members agree to cooperate with survival follow-up.

Exclusion Criteria

1. Have multiple factors affecting oral medications (such as inability to swallow, chronic diarrhea, and intestinal obstruction); 2. Have a history of hemorrhage, any serious grading of 4 degrees or more in CTCAE 4.0 within 4 weeks before screening; 3. Patients with high blood pressure who are not well controlled by a single antihypertensive drug (systolic blood pressure > 140 mmHg, diastolic blood pressure > 90 mmHg); those with a history of unstable angina pectoris; newly diagnosed with angina within 3 months prior to screening Cardiac infarction occurred within 6 months before screening; arrhythmia (including QTcF: male ≥ 450 ms, female ≥ 470 ms) requires long-term use of antiarrhythmic drugs and New York Heart Association grade ≥ grade III cardiac insufficiency; 4. Urine routine indicates urinary protein ≥ +++ and confirmed 24-hour urine protein quantitation > 4.0 g; 5. Long-term unhealed wounds or incompletely fractured fractures; 6. Imaging shows that the tumor has invaded the important perivascular circumference or that the patient's tumor has a high probability of invading the important blood vessels during the treatment period and causing fatal bleeding; 7. Coagulation dysfunction, with bleeding tendency (must be satisfied 14

days before randomization: INR is within normal range without anticoagulant); anticoagulant or vitamin K antagonist such as warfarin, Patients treated with heparin or its analogues; small doses of warfarin (1 mg orally, once daily) or low-dose aspirin are allowed for prophylactic purposes, provided that the international normalized ratio (INR) of prothrombin time is ≤ 1.5. Do not exceed 100 mg per day); 8. For female subjects: should be surgical sterilization, postmenopausal patients, or agree to use a medically approved contraceptive during the study treatment and 6 months after the end of the study treatment period; before the study was enrolled The serum or urine pregnancy test must be negative within 7 days and must be non-lactating. Male subjects: should be surgically sterilized, or consent to use a medically approved contraceptive during the study treatment and within 6 months after the end of the study treatment period. 9. Persons with a history of psychotropic substance abuse who are unable to quit or have a mental disorder; 10. Have a history of immunodeficiency, or have other acquired, congenital immunodeficiency diseases, or have a history of organ transplantation; 11. According to the investigator's judgment, there are serious concomitant diseases that compromise the safety of the patient or affect the patient's completion of the study.
